# Comparative Aspects of Annelid Regeneration: Towards Understanding the Mechanisms of Regeneration

**DOI:** 10.3390/genes12081148

**Published:** 2021-07-28

**Authors:** Roman P. Kostyuchenko, Vitaly V. Kozin

**Affiliations:** Department of Embryology, St. Petersburg State University, Universitetskaya nab. 7-9, 199034 St. Petersburg, Russia; v.kozin@spbu.ru

**Keywords:** invertebrates, annelids, dedifferentiation, stem cells, wound healing, blastema induction, blastema origin, segmentation, nervous system, digestive system, gene expression, transcriptomics, evolution

## Abstract

The question of why animals vary in their ability to regenerate remains one of the most intriguing questions in biology. Annelids are a large and diverse phylum, many members of which are capable of extensive regeneration such as regrowth of a complete head or tail and whole-body regeneration, even from few segments. On the other hand, some representatives of both of the two major annelid clades show very limited tissue regeneration and are completely incapable of segmental regeneration. Here we review experimental and descriptive data on annelid regeneration, obtained at different levels of organization, from data on organs and tissues to intracellular and transcriptomic data. Understanding the variety of the cellular and molecular basis of regeneration in annelids can help one to address important questions about the role of stem/dedifferentiated cells and “molecular morphallaxis” in annelid regeneration as well as the evolution of regeneration in general.

## 1. Introduction

Regeneration is an everyday physiological process or an immediate response to traumatic injury after amputation or autotomy. It occurs at different developmental stages, from embryos and larvae to full-grown adults. Although virtually all animals are capable of physiological regeneration (periodic replacement of cells and tissues, e.g., gut or epidermal tissues), the ability to regenerate lost body parts or the entire body (restorative, or post-traumatic regeneration) varies widely, sometimes even between closely related species [1,2,3,4].

Post-traumatic regeneration is a complex process. It includes several partially overlapping main events such as:

- closure and wound healing;
- immune and/or nonspecific defense reaction;
- recruitment of cells that are to form a regenerate;
- growth of the regenerate;
- patterning and differentiation.

The duration of each phase can vary greatly depending on the anatomy of the object, the nature of the wound, and the place and method of amputation. However, each of these phases is accompanied by a change in gene expression. Taken together, these events lead to the induction of the regeneration process, including reprogramming of dormant stem and/or somatic cells, cell proliferation, and/or remodeling of surviving tissues [3,5].

Post-traumatic regeneration has been recorded in many phyla of invertebrates such as cnidarian, flatworms, annelids, mollusks, arthropods, echinoderms, and ascidians, etc. [2,3,6,7,8]. Annelids (segmented marine, freshwater, and terrestrial worms) are capable of regeneration at various levels of their biological organization, including cell repair, germ cell restoration, regrowth of structures, or the regeneration of the entire body from small fragments [9,10,11,12]. 

Restorative regeneration in annelids does not necessarily result from injury; many of them have marked capacities for regeneration associated with asexual reproduction [2,4,13,14,15]. The most remarkable abilities to regenerate the head and the “tail” segments lost due to injury are called, respectively, anterior and the posterior regeneration. Both these types of segmental regeneration are ancestral for annelids. However, they have also been lost several times in the course of evolution (18 and 5 times, respectively), and never regained [15,16]. Some annelid species (leeches, dinophilids, sandworm *Arenicola marina*) are either completely incapable of segmental regeneration, or they demonstrate a very limited regeneration [6,9,10,17]. On the other hand, even among species that can regenerate the anterior end, the degree of this ability often varies depending on the position of the amputation site along the anterior–posterior axis. For example, in the earthworm *Eisenia foetida* and the marine annelid *Autolitys pictus*, anterior regeneration is significantly reduced as more segments are removed. These species are unable to regenerate the anterior end after the number of anterior segments removed exceeds a certain limit [6,18].

Regeneration of most annelids occurs by epimorphosis, involving cell proliferation and the formation of a blastema, a mass of undifferentiated cells. During epimorphosis, cell divisions are activated, which leads to the formation of a regenerative bud. Due to the cellular material of the regenerative bud, the lost tissues and organs are restored in accordance with the original structures [10]. However, epimorphic regeneration is always accompanied by morphallaxis, i.e., by remodeling of the existing tissue [19]. 

Anterior and posterior regeneration in annelids may differ in many characteristics such as the size of the regeneration buds, origin of blastema cells, time of duration, patterns of segmentation, and differential gene expression [6,9,10,11,12,20,21,22]. The anterior regeneration bud is typically larger than the posterior bud. During posterior regeneration, segment restoration appears to involve mechanisms similar to those during normal posterior segment addition (a posterior growth zone is established and then segments are added sequentially), but occurs at a greatly accelerated rate. In contrast, anterior segments appear simultaneously during anterior regeneration.

The diversity of regenerative capabilities makes annelids an excellent model for comparative studies of regenerative mechanisms. Here, we review many recent studies on molecular, cellular, and tissue level processes that occur during regeneration in annelids. Despite a long history of research on annelid regeneration, many important questions regarding its fundamental mechanisms remain unsolved. In particular, it is still unclear why annelids are capable of regeneration at various levels of their biological organization and can regrow a complete body from small fragments.

## 2. The Transcriptomic Era

A detailed analysis of gene activity during regeneration can help to reveal the mechanisms promoting or inhibiting this process. It can also help to address important issues such as the relationship between regeneration, asexual reproduction and embryonic development, and the evolution of regenerative processes within the Annelida and across the animal kingdom.

In the last few decades, the expression of genes involved in regeneration in annelids has been mainly analyzed with the use of in situ hybridization (see below). The results of these studies are very important because they help researchers to interpret the regenerative processes obvious at the morphological level. However, in situ hybridization does not provide bulk data. The study by Myohara et al. [23] on *Enchytraeus japonensis* was the first large analysis of gene expression during annelid regeneration. Using a subtraction cloning method, the authors identified 165 cDNAs upregulated during regeneration, including 79 sequences encoding known factors and 86 sequences representing putatively unknown genes. A wide range of proteins was found among the former, such as metabolic enzymes, components of extracellular matrix, cytoskeletal proteins, transcription factors, putative receptors, and RNA helicase. The following high-throughput expression profiling analyses performed on *Perionyx excavatus* [24], *Pristina leidyi* [25], *Eisenia fetida* [26,27], *Sphaerosyllis hystrix* and *Syllis gracilis* [22], *Eisenia andrei* [28], *Eudrilus eugeniae* [29], and *Lumbriculus variegatus* [30] have lent support to the idea that a large and diverse repertoire of genes are up-regulated or down-regulated during regeneration in annelids. Many studies have shown the activation of, not only housekeeping genes and components of signaling pathways (FGF, MAPK, BMP, WNT, Hedgehog, Notch), but also genes that have specific functions in development, including homeobox genes (*Hox, homothorax, even-skipped, msx*), *brachyury*, factors related to nervous system development (*gs, elav, slit*), and stem-cellness [22,26,29,30]. 

However, in most studies, only one type of regeneration has been examined, even if the animals in question could regenerate both anterior and posterior body segments quite effectively. The mixed total RNA of different regeneration stages has been often used for the transcripts analysis, without comparison of the regenerating tissue with the adjacent control tissue. Moreover, many studies focused on the transcriptional activity of *a priori* selected candidate genes. The differences in the stages of regeneration used in different works also decrease the value of the published results for comparative transcriptomics (see Table 1).

Nevertheless, the transcriptomic approach, particularly that based on the identification of differentially expressed genes (DEG), is very helpful for identification of a common genetic program required for regeneration in annelids and other animals. It may also be used to discover new candidate genes involved in regenerative processes. Thus, homologues of regeneration-related genes, *Ej-rup 1–5* [23], were identified in the transcriptomes of two syillid species. In *S. hystrix*, *Shy-rup2* was upregulated during anterior regeneration. Although the functions of this gene are unclear, the expression of *Ej-rup2* in epidermal blastema cells during anterior regeneration in *E. japonensis* suggests its regeneration-specific role [22,23].

In a recent work by Ribeiro et al. [22], a large number of genes with differential expression during regeneration was detected in two syllid species (Table 1). Both species can completely regrow the posterior end, but only *S. gracilis* can regenerate the entire anterior end, whereas *S. hystrix* shows an incomplete anterior regeneration. The same level of differential expression has been shown for *E. fetida* [26] and *E. andrei* [28]. Similarly to other animals [5], the highest level of DEG is observed at the earliest stages of regeneration, but most of the detected DEG are specific to anterior regeneration. The results of comparative transcriptomics support the opinion [6,21] that posterior regeneration is similar to the post-larval growth in annelids, while anterior regeneration is markedly different [22]. 

Despite the importance of the transcriptome approach, there are still very few data for comparative transcriptomics. On the other hand, the bulk data on gene expression have been obtained by different research strategies, often without regard to the mechanisms of regeneration at the cellular and the tissue level or the variability of the regeneration among annelids. Obviously, it is impossible to tackle the questions associated with regeneration in annelids without paying close attention to morphological aspects and standardization of comparative transcriptomics studies.

## 3. Back to the Future

### 3.1. Early Events during Regeneration: Wound Healing and Immune Response

Despite the epimorphic character of the post-traumatic regeneration in annelids, i.e., the formation of a mass of actively proliferating undifferentiated cells, pre-existing tissues are essential and often critically important from the earliest stages of this process. Firstly, they are involved in wound closure and immune response. Secondly, they are a source of factors inducing further regeneration processes. Thirdly, they are a source of cells that repair damaged tissues and redevelop lost body parts and, at the same time, signals that allow these cells to be recruited.

**Figure 1:**
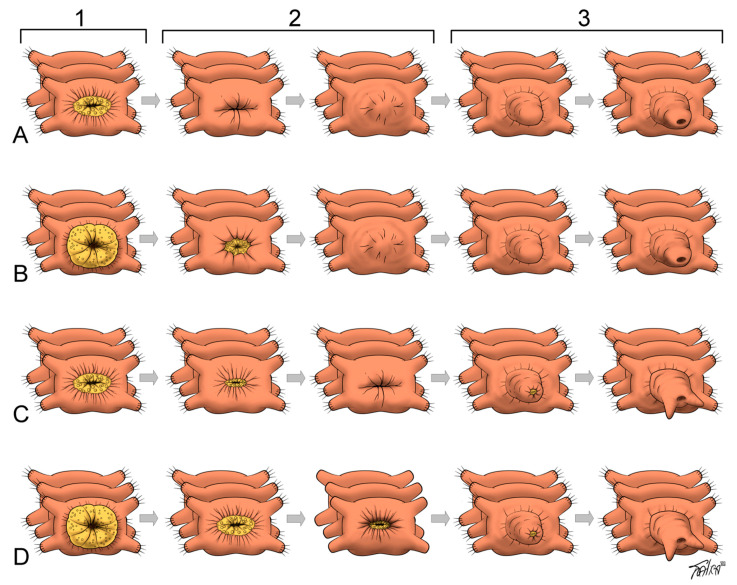
Variability and staging of large-scale regeneration events in annelids after anterior (**A**,**B**) or posterior (**C**,**D**) amputation. The schemes illustrate changes at the amputation site (an intersegmental transverse cut). (**A**,**B**) Variants of complete closure of the gut opening by the wound epithelium. Oral or anal opening forms by an independent invagination of the superficial epithelium. These variants are characteristic of the anterior regeneration in all annelids tested as well as of the posterior regeneration in oligochaetes and most polychaetes. (**C**,**D**) During the “open regeneration”, the intestinal epithelium joins the epidermis, so that the gut lumen is always connected to the external environment. These variants are characteristic of the posterior regeneration in some polychaetes, such as nereids, sabellids, *Harmothoe*, and *Nephtys*. Column (1), an immediate response to amputation: primary wound closure is ensured by (**A**,**C**) constriction of circular muscles, which may be accompanied by (**B**,**D**) intestinal protrusion (yellow), forming a primary wound-plug. Column (2), stages of epithelialization and blastema initiation. Column (3), growth and differentiation of regenerative bud. The horn-like structures in (**C**,**D**) illustrate formation of pygidial cirri, as seen in nereids (Figure 4D).

Following segment amputation, the wound closes with the formation of wound epithelium. Wound closure begins immediately after amputation. It occurs mostly by rapid muscle contraction proximal to the amputation plane. A slight extrusion of the severed gut sometimes contributes to the sealing of the wound [4,6,10,12,17]. If an animal cannot close the wound, it dies [9,17]. Initial muscle contraction is followed by the migration of coelomic cells to the wound site where they form a wound plug and later by reepithelialization (reviewed [6,10], Figure 1). 

**Figure 2:**
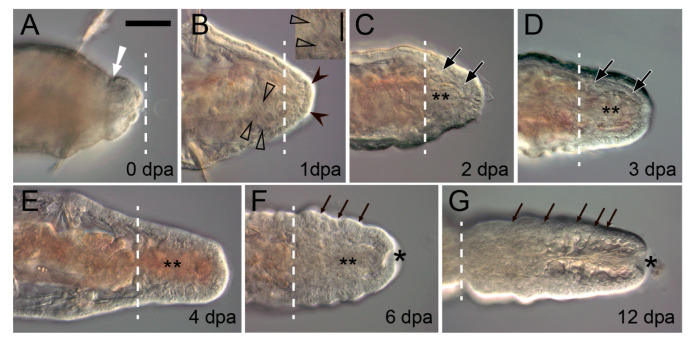
Posterior regeneration in the juvenile oligochaete *Tubifex tubifex* (Clitellata, Naididae). Optical frontal sections of live anesthetized specimens 0 to 12 dpa; posterior end is to the right. Dotted line, plane of amputation; double asterisk, gut lumen; white arrow in (**A**), constriction of circular muscles; triangles in (**B**), numerous large spindle-shaped cells with a large nucleus and a nucleolus at the end of cut VNC (higher magnification view on the insert); black arrowheads in (**B**), wound epithelium covering the whole stump; large arrows in (**C**,**D**), mesodermal cells of the regeneration blastema, which is already segmented at 4 dpa (**E**); asterisk in (**F**,**G**), anus; small arrows in (**F**,**G**), segmental boundaries. Scale bar in (**B**, insert) 20 μm, in all other panels 50 μm.

The movements of cells to the wound appears to be a general feature of annelids (Figure 2). Extensive cell migration occurs within minutes to hours after amputation, but it can be usually observed during the first day after injury and even longer [31,32,33]. Migrating cells not only generate the wound plug by clotting and may contribute to the regenerating structures, but also provide an innate immune response. Coelomocytes migrate to the wound site and phagocytize cellular debris, including damaged epithelial and muscle cells [6,10]. The expression of immune response-related genes during annelid regeneration is still poorly characterized. Kinetical changes in the mRNA patterns of PRRs (pattern recognition receptors TLR, CCF, LBP/BPI, SR) and AMPs (antimicrobial peptides lysenin, lumbricin, and lumbricin-related peptide) have been recently shown for regenerating earthworm *E. andrei* [33]. Increased mRNA expression of CCF, an earthworm-specific PRR that recognizes a broad range of pathogen structures, was found in the early anterior blastema. In the posterior blastema, its expression decreased at two weeks, but slightly increased later, at the four-week stage. The level of LBP/BPI and AMPs expression was lower throughout the anterior and the posterior regeneration. Thus, the expression of the immune response-related genes was decreased in regenerating *E. andrei* as compared to intact earthworms. This phenomenon, which is not unique for earthworms, may indicate that the blastema is an immunologically tolerant niche ensuring successful regeneration [33].

After anterior and usually after posterior amputation, the wound heals by fusion of the severed edges of the epidermis over it. In this type of healing, the severed gut edges also fuse, and a blind gut end forms [10,12]. In some cases of posterior regeneration, the severed edges of the epidermis and the gut epithelium fuse directly with each other and restore the anus by the wound healing process (“open regeneration” in nereids [34]).

The first events after injury are marked by significant changes in cell–cell adhesion and the extracellular matrix. These changes may play a key role in the next step of regeneration, as shown for many other phyla [5,10,35]. Extensive studies, following anterior amputation in annelids, mostly performed on histological sections, showed modifications of the cellular matrix, the absence of a basement membrane close to the wound epithelium, and the loss of cell contacts. All these changes may activate the cell dedifferentiation and proliferation in adjacent tissues [10,32,36,37]. Immediately after amputation, there is also a short period of downregulation of cell divisions, which may be local or body-wide [6,10,12,38,39,40,41,42,43]. The wound epithelium is formed by the rearrangement of epithelial cells, but not by their active proliferation [36,37,38,39]. Transition to the next stage of regeneration, the blastema formation, is characterized by a sharp upregulation of cell proliferation and several genes known to be important developmental regulators [12,34,39]. 

### 3.2. Cellular Sources of the Blastema

It is generally accepted that cells of all three germ layers contribute to the blastema; however, specific cellular sources may vary in different annelids [4,6,10,43]. While the studies of the regeneration in many other animal groups concentrated on just one or two model systems, the cellular origins of the blastema have been examined in a broad range of annelid species. Conclusions regarding the cellular aspects of annelid regeneration are mostly based on the analysis of fixed tissues using microscopy, histology, in situ hybridization, and proliferative markers assays, combined with experiments with different strategies. Although the results of these studies are very valuable, they do not provide direct evidence and need to be verified with modern methods of in vivo cell tracing, which are still poorly developed for annelids [6,10,11,12]. According to the literature, the presence of somatic stem cells has been shown for some species. On the other hand, many studies have demonstrated a dedifferentiation of cells and their subsequent reproduction. The restoration of mesodermal structures due to dedifferentiated ectodermal derivatives has been reported in older works [10,44]. According to the results of a histological study of *Owenia fusiformis* regeneration, myocytes dedifferentiate and begin to divide, forming the missing muscle elements [36]. Restoration of the nerve ganglia by epithelial cells migrating inwards has been reported for *Limnodrilus hoffmeisteri*, *Ophidonais serpentina*, and *L. inconstans* [31,44] (see also below).

It is known that dedifferentiation and subsequent proliferation of cells occur only in response to amputation, while reserve stem cells maintain their proliferative activity all the time. This means that the nature of the cells that contributing to the regenerated structures can be studied with the use of proliferation markers such as EdU and BrdU. In *Syllis malaquini*, as recently shown with double EdU-chase/BrdU-pulse treatments, the blastema consists exclusively of cells that enter the S-phase after injury. These results suggest that the blastema is formed by cell dedifferentiation and redifferentiation, although it cannot be completely ruled out that stem cells are also involved in regeneration [45]. Once formed, the blastema shows a high proliferative activity of its cells, which has often been confirmed by using the DNA precursors [38,39,40,41,42]. In *Platynereis dumerilii*, it was also documented by in situ hybridization with *cyclin* and *pcna mRNA* probes, because the protein products of these genes are involved in DNA replication. In addition, the inhibition of proliferation at the biochemical level led to a disruption of the formation of the regenerative bud [39]. A more targeted study demonstrated a critical role of FGF signaling in the initiation of proliferation in *Alitta virens* after amputation [46].

Germline multipotency program (GMP) genes are strong molecular markers of the multipotent state of cells [47]. Many studies have shown the involvement of the GMP genes in the processes of regeneration [16,39,40,41,42,48]. Moreover, a correlation of the failure of anterior regeneration with the absence of *nanos* expression has been reported for some naidid annelids [16]. De novo expression of these genes in the blastema may indicate local dedifferentiation, while the detection of cells expressing GMP in intact segments may indicate the possibility of stem cell migration. Analysis of GMP gene expression and the study of cell proliferative activity during regeneration may help one to reveal probable cellular sources. It has been shown using a combination of these techniques that the posterior blastema of *P. dumerilii* consisted of dedifferentiated cells [39]. To identify stem elements and their possible migration, the authors incubated the worms in EdU prior to amputation. Most of the blastema cells were unlabeled, indicating that the migrating cells did not contribute to the formation of the regenerate. GMP gene expression (*piwi*, *vasa*, *nanos*, *pl10*) was detected only in the blastema cells, which also suggested that the blastema development was driven by cell dedifferentiation processes [39].

The dedifferentiation of cells adjacent to the wound was also shown for other annelids [41,43,48]. During the anterior and the posterior regeneration of *P. leidyi*, *de novo* expression of the GMP genes *vasa*, *piwi*, and *nanos* occurs in both epidermal and deep cells of the regenerate [48]. The authors point out the absence of cells with a similar expression profile in intact segments, which is consistent with the idea of dedifferentiation. A recent study on the marine annelid *A. virens* has also clearly demonstrated de novo expression of GMP genes *vasa*, *piwi*, and *pl10* during posterior regeneration [41]. 

In contrast, migrating cells are involved in posterior regeneration in *Capitella teleta*. A possible cellular source of *vasa*-positive cells migrating to the wound is a heterogeneous cluster of multipotent progenitor cells (MPC), which appears during embryogenesis. Following amputation at the level posterior to the MPC, these worms restore up to 12 posterior segments, but when the cluster is removed, they lose this regenerative ability. Based on these results, the authors hypothesize that the cells of the MPC cluster migrate to the injury site and contribute to the blastema during posterior regeneration in *C. teleta* [40].

The phenomenon of cell migration during regeneration has been most thoroughly studied in clittelate annelids. The movement of specific spindle-shaped cells, which appear to be undifferentiated or weakly differentiated, was first described in the fixed tissues of regenerating *Lumbriculus* by Randolph [49], who called them neoblasts (this term is now widely used to refer to the unique flatworm pluripotent stem cells [50], though there is no evidence of a homology between annelid neoblasts and planarian neoblasts). Later, neoblasts have been described in other species such as *L. hoffmeisteri*, *Ophidonais serpentina*, *E. japonensis*, and *P. leidyi* [31,42,44]. Based on time-series of fixed material, the authors concluded that these cells originated from the base or the surface of the septa, were activated following amputation, and migrated to the injury site typically along the ventral nerve cord (VNC). The search for these cells in other annelids failed. Neoblasts migrating across the body appear to be restricted to the Clitellata [10].

In the oligochaete *P. leidyi*, the presence of annelid neoblasts was confirmed with the use of 4D microscopy [51]. During posterior regeneration, neoblasts migrate along the VNC, while during anterior regeneration, they move along the lateral walls of the body. In addition, it was shown for the first time that neoblasts-like cells could also migrate from the blastema. These results expand the range of potential functions of neoblasts [51]. On the other hand, migration of cells from the blastema may be evidence of a random-walk behavior of these cells. Interestingly, despite an active cell migration during regeneration, GMP genes (*vasa*, *piwi*, *nanos*) are expressed de novo during anterior and posterior regeneration, which indicates the absence of migrating cells with the same expression state. This means that regeneration occurs due to a local dedifferentiation of cells. 

On the other hand, neoblasts expressing *vasa* were found in the oligochaete *E. japonensis* [42,52]. These cells are located near the septa in each segment of the body, starting from the seventh and further to the posterior end. Following amputation, they divide and migrate to the wound site, forming the inner part of the blastema [53,54]. The authors suggested that only neoblasts were involved in the regeneration of the mesodermal derivatives. However, the results of the experiments with EdU suggest that the regrowth of the gut and the epidermal structures occurs due to the derivatives of the ectoderm and the endoderm, respectively. The anterior segments of *E. japonensis* that do not contain neoblasts retain the ability to restore the lost posterior end of the body, which means that other cellular sources are involved in this process. Neoblasts probably provide the specific information to restore the correct number of head segments. If more than seven anterior segments are amputated (in this case, there are neoblasts in the segment closest to the wound), only seven head segments are always restored, regardless of where the cut was made. However, when less than seven segments are removed (there are no neoblasts in the segment nearest to the wound), only the number of amputated segments are regenerated. According to the authors, it is the neoblasts that are responsible for the fine-tuning of the regeneration process [53,54].

Interestingly, despite targeted studies, neoblasts have never been found in a closely related species, *E. buchholzi*, which is nevertheless capable of regeneration [55]. In contrast to *E. japonensis*, the number of restored anterior segments in *E. buchholzi* correlates with the amputation site: the closer to the posterior end of the body an operation was made, the fewer segments were restored. This is an additional confirmation of the idea that neoblasts carry positional information, rather than being the main source of cells for regeneration. It was shown with the use of BrdU incorporation experiments that the activation of the proliferation activity of the blastema cells in *E. buchholzi* occurred only by the fourth day of regeneration, while *E. japonensis* forms a large blastema as early as on the first day of regeneration. Probably, neoblasts induce the proliferative activity of the blastemal cells. Similarly to some other types of migrating cells, they may affect the rate of cell proliferation in the regeneration bud. For example, experiments on coelomocyte depletion have shown a significantly decreased cell proliferation in both anterior and posterior blastema in *E. andrei*. As a result of the ablation of coelomocytes, the animals failed to complete the regeneration of the lost body parts within four weeks [33]. In light of the discovery of the new possible functions of neoblasts, the question about the cellular sources of blastema remains open.

Though there is a large body of valuable older literature on the cellular basis of regeneration in annelids (reviewed by Bely [10]) as well as a number of more recent studies, the cellular sources of this process are not fully understood. It is known that they may differ for anterior and the posterior regeneration, even within the same species [11,12,56]. For example, in *L. variegatus*, the formation of the posterior blastema is suppressed by pharmaceutical inhibition of cell migration, while a small anterior regenerative bud is nevertheless formed under the same conditions. Thus, posterior regeneration in these animals can be fully provided by migrating cells, while anterior regeneration is ensured by both migration and local dedifferentiation [57].

At the blastema differentiation stage during posterior regeneration, GMP gene expression is observed in the cells of the newly formed posterior growth zone. In modern literature, it is customary to separate the posterior growth zone (PGZ) and the segment addition zone (SAZ). The PGZ represents the entire prepygidial region where segment formation occurs. SAZ is a part of PGZ and consists of a narrow ring of stem cells. The descendants of these cells give rise to the new segments. The stem state of cells in SAZ has been confirmed in several studies [39,40,58]. The expression of tissue-specific genes by the cells of the posterior part of the blastema, even before isolation of the growth zone, is evidence of the multipotent rather than pluripotent state of the cells in SAZ [39]. The hypothesis that SAZ is a stem niche is also supported by a more intense expression of GMP genes in that zone than in the rest of the PGZ. However, this classification is not generally accepted, and some authors use only the term "posterior growth zone", without identifying a separate zone of addition of segments.

During posterior regeneration of annelids, two phases of GMP gene expression can usually be distinguished: blastema-wide expression until the beginning the blastema differentiation stage and a later expression mostly restricted to the PGZ-cells [39,48,59]. The GMP expression pattern in PGZ varies in different annelid species. Expression of *piwi*, *vasa*, and *nanos* homologues was shown in developing PGZ during regeneration in annelid *P. leidyi* [48]. In this case, *piwi* was expressed more broadly, while *vasa* and *nanos* transcripts were found only in the SAZ-cells. During the regeneration of *C. teleta* worms, the PGZ-cells accumulated *piwi* transcripts [59]. In regenerating *P. dumerilii*, *piwi*, *vasa*, *pl10*, and *nanos* homologues were expressed in the PGZ [39]. In the oligochaete *E. japonensis*, PGZ is characterized by the expression of *vasa* homologues [53]. On the contrary, *vasa* expression is absent in the PGZ of the oligochaete *Tubifex tubifex* [60]. In that case, the absence of the protein product of this gene could be compensated by the related RNA helicase *p68* [61].

During anterior regeneration, GMP gene expression has been shown only in *E. japonensis* and *P. leidyi* [42,48]. In *P. leidyi*, the anterior blastema is characterized by *piwi*, *vasa*, and *nanos* homologue expression. In *E. japonensis, vasa*-positive cells in the anterior blastema have also been described. 

Thus, GMP genes are involved in the anterior and posterior regeneration in annelids. They mostly start to be expressed de novo, although they are differentially expressed within the regeneration bud, and the patterns of their expression vary across the annelids studied in this respect. This consideration and the results of the EdU/BrdU experiments suggest that the blastema cells originate mostly locally, probably by dedifferentiation of the cells at the wound site in many species. On the other hand, some long-distance migrations of source-cells are documented in several species. However, participation in the blastema formation may not be the only function of these migrating cells. They might also be involved in the induction and patterning of the blastema via positional information (see also below).

### 3.3. Regeneration of Specific Tissues

#### 3.3.1. Nervous System 

Most annelids have significant abilities for the regeneration of the nervous system (NS). After amputation, elements of the central nervous system (head and abdominal ganglia), as well as peripheral nerves, are fully restored as parts of regenerating segments [12,21,62,63,64]. In this scenario, there is no significant degeneration of the nervous tissue of the old segments. At the same time, after the transection of the VNC and the removal of individual neurons and ganglia, these elements of the nervous system can be replenished to varying degrees [65,66,67]. Oligochaetes employ the fastest and most efficient way to restore the integrity of the VNC after transection, removal of its fragments, or transplantation [68,69,70,71]. Leeches have the least potential for neural regeneration, and only the restoration of neural connections (synapses) and reinnervation have been described [67,72,73,74,75,76]. Regenerative events in leeches are more autonomous than embryonic neurogenesis. For example, when axons are being repaired, their growth stops where the synapse was previously located, not at the initial target [77]. Apparently, this overall variability is associated both with the synapomorphies of each large taxon and with physiological limitations that have arisen adaptively in different species of annelids.

**Figure 3:**
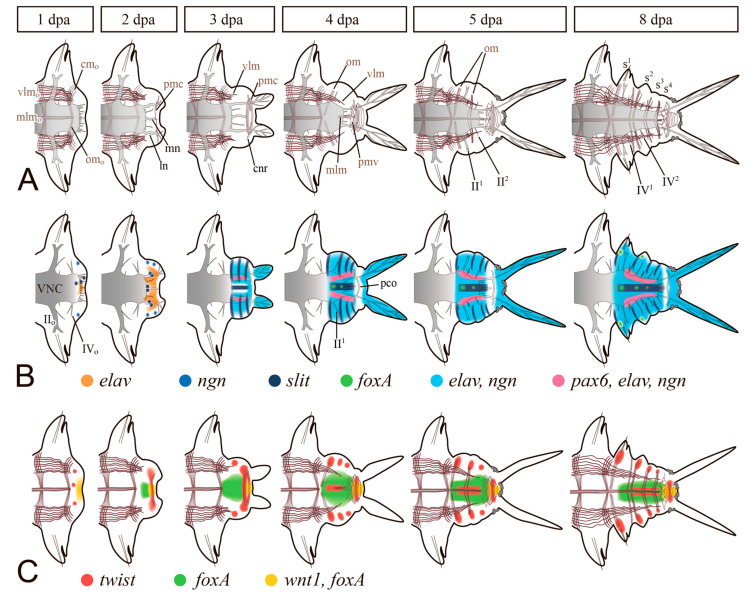
Dynamics of muscle and neural regeneration combined with corresponding gene expression patterns in nereid polychaetes (*Alitta virens* and *Platynereis dumerilii*). Ventral views and posterior body end (regenerative bud) is to the right. (**A**) Morphological illustration of muscles (brown) and nerves (grey fill) development. (**B**) Expression patterns of *elav*, *neurogenin* (*ngn*), *slit*, and *pax6* in neurogenic tissues. (**C**) Expression patterns of *twist* in muscle precursors and *foxA* and *wnt1* in intestinal tissues. **II**, **IV**, segmental nerves of VNC; **cm**, circular muscles; **cnr**, circumpygidial nerve ring; **ln**, VNC lateral nerve; **mlm**, ventral median longitudinal muscle; **mn**, VNC median unpaired nerve; **om,** oblique muscles; **pco**, pygidial commissure; **pmc**, pygidial circular muscles; **pmv**, network of ventral muscles in the pygidium; **s**, segments with external furrows; superscript symbol, serial number of regenerated segment, which possesses the structure, defined by the letter; subscript o in **II_o_**, **IV_o_**, **cm_o_**, **mlm_o_**, **om_o_**, and **vlm_o_** denotes affiliation of nerves and muscles to the old tissues of the stump. Modified after [12,34,39,79].

Regeneration of the NS after the amputation of segments involves both nearby tissues and newly formed cells of the regenerative bud (Figure 3). First of all, neurites from the VNC and the peripheral nervous system grow into the wound site and penetrate the wound epithelium [38,39,64,78,79]. Concurrently, the nerve plexus forms around the intestine. Although the first stages of wound healing can occur in the absence of innervation [80,81], the neurites are crucial for the initiation and growth of the blastema and morphogenesis [20,65,82]. By the time the regeneration blastema appears, the neurites may form a different number of more or less thick nerves, terminating at the site of pygidial or prostomial appendages formation [78,79,83,84]. Lateral nerves of the VNC often join in a loop, which is typical of anterior regeneration [85,86,87,88,89,90] but also occurs in the case of posterior regeneration. From this loop, a circumesophageal nerve ring forms in the anterior regenerate or a circumpygidial nerve ring in the posterior regenerate (Figure 3). The neural precursor cells of the regenerative bud concentrate around the primarily formed neuropil. Although the origin of these cells has not been traced with certainty, they probably ingress from the superficial epithelium of the bud. The anterior dorsal part of the regenerate is considered to be the source of the brain, and VNC is presumably formed from the ventrolateral zones [12]. Distinct ganglia emerge during segmentation of the regenerative bud in polychaetes (Figure 3), this process having a prominent anterior-to-posterior gradient in the posterior regeneration [91]. Thus, several conservative features can be traced in the epimorphic recovery of NS.

Along with an active remodeling of the VNC stump near the wound site, significant rearrangements occur throughout the entire pre-existing (old) NS. The phenomenon of the neural morphallaxis has been described in detail using evidence from the regeneration of *L. variegatus* [92,93,94]. Because a fixed number of anterior segments is always restored in *Lumbriculus*, the fragment of the old body located just posteriorly to the wound undergoes anteriorization. Neural morphallaxis is based on synaptic plasticity, i.e., changes in the functional status of neural connections [95]. Rapid changes in the neural networks during the first days after injury lead to a reorganization of sensory fields in accordance with their new position in the body. This is manifested by a switch in the behavioral response to stimulation, which differs in the case of the anterior and the posterior part of the body. Behavioral changes are also accompanied by structural and functional transformations of the VNC. The diameter of giant axons and the abundance of gap junctions, as well as the speed of nerve impulse conduction, changes according to the new positional values within a few weeks after amputation [96]. Surprisingly, the epimorphic component of *Lumbriculus* regeneration is not required for the induction of morphallaxis in neural networks. However, the progress of epimorphosis is closely connected with the long-term consolidation of the subtle mechanisms of neural morphallaxis [97].

The molecular basis of neural regeneration has been poorly investigated. Works on the unbiased analysis have identified both conservative and novel potential drivers of reparative neurogenesis [22,23,26,27,28,98,99]. Among the NS-associated genes, the strongest differential expression was noted for such neurodifferentiation markers as *glutamine synthetase, slit, elav, neurofilament NF70,* and *Nerve Growth Factor* [22,23,26,28,39,100]. Spatiotemporal dynamics of gene expression in the regenerating NS has been documented only in nereid polychaetes (Figure 3B) [39,41,46,91,101] and *C. teleta* [78]. Among the genes demonstrating the earliest and the most drastically rearranged expression are *Lox5*, *Lox2*, *Post2* [91,101], *pl10* [41], and *FGF*s and *FGFRs* [46]. All these markers activate their expression in the VNC ganglia adjacent to the wound in the first hours after amputation. The role of this repatterning, which we refer to as “molecular morphallaxis” [19], is, apparently, to update positional information. This is confirmed by the expanding activation of the genes mentioned above in the epimorphic regenerative bud, which has the most posterior axial value. Later on, the nascent neurogenic tissue launches the expression of *Hox5*, *Hox7*, *Hox1*, *Hox4*, and *Lox4* [91,101]. The Hox code is much more stable during regeneration of *C. teleta* [78], although the authors noted a limited morphallactic shift in the expression domains of *Lox4*, *Lox2*, and *Post2*. The expression of some neural markers is observed in *P. dumerilii* already at the early stages of wound healing [39]. Although an unambiguous determination of cell identity in the expression domains is not always possible, their localization indicates the reorganization of the wounded tissues involved in neurogenesis. The expression areas of *neurogenin*, *slit*, *elav*, and *pax6* are found mainly on the ventral side of the bud, as well as in the pygidium anlage [39]. Strikingly, the order of the proneurogenic genes activation during regeneration is not identical to that during the larval development of the neuroectoderm in *Platynereis* [12].

The earliest studies on annelid postembryonic development revealed the leading role of the NS in segments regeneration [102,103,104,105,106,107]. Experiments on denervation, transplantation, transection, and deviation of the VNC reinforced the suggestion that its damaged end is required and sufficient to induce the development of a regenerative bud [6,20,21,62,108,109,110,111]. The NS in annelids is currently believed to have, not only trophic and organizing functions, but also a vital role in creating positional information that controls the specificity of morphogenesis [10,19,112,113]. In addition to its involvement in maintaining anterior-to-posterior polarization (see above), VNC has an undoubtedly essential role in ventralization of the body wall [6,10]. In experiments on VNC deviation, regeneration is either completely blocked or results in a radially symmetric regenerate, in which all sides have a dorsal identity [106,107,114].

Despite ample experimental evidence of NS involvement in annelid regeneration, the specific mechanisms of its influence remain a mystery. In theory, there are three aspects of its involvement in regeneration events. First of all, nerves can be a local source of signaling molecules, such as various growth factors, BMPs, substance P, AGP, and transferrin. The effect of these secreted factors on the stimulation of cell division and the development of the regenerate has been well described for the model of vertebrate limb regeneration [115,116,117]. The repertoire of these molecules in annelids is poorly characterized, but some pioneering data suggest their relevance for regeneration [46,118,119], and so do the observations on the suppression of proliferation in a denervated wound [10,40,82]. Neurotransmitters are an important NS-derived factor, but their role in annelid regeneration remains almost unexplored [120,121]. The identification of specific neurotrophic factors is clearly one of the most promising directions for understanding the causality of annelid regeneration. Apparently, given the trophic and organizing role of the NS, the specificity of these factors should vary at different regeneration stages.

The second possible regulatory aspect of the NS is its accessory role for other cell types participating in the regenerative response. The migratory elements temporarily associated with the NS are phagocytes, neoblasts, and germ line cells, as described in some annelids, primarily in oligochaetes [48,51,53,55]. Subtle interactions between the NS and regenerate precursor cells remain to be uncovered. Probably, the interactions of cells and growth factors with the extracellular matrix have an important role here [3]. 

The third aspect is the striking and, in many respects, unique role of the systemic regulation of annelid regeneration and sexual maturation through neuroendocrine secretion [122,123,124]. In many annelids, gametes maturation is activated after an ablation of the cerebral brain (the source of the hormone), and the segment regeneration becomes impossible. However, this effect may be manifested in opposite ways during different ontogenetic stages and in different taxa [65]. Recent advances in the studies of the molecular nature of such humoral factors in nereids and syllids [125,126,127] open fascinating possibilities for a fundamentally new understanding of the NS systemic action on specific targets in the context of environmental conditions.

The available data indicate that the details of neural regeneration of annelids are crucial for understanding the general mechanisms of the injury response. Considering the high regenerative abilities of annelids, this model becomes increasingly more popular. Future research should focus on a comprehensive description of the morphological, molecular-genetic, regulatory, systemic, and evolutionary patterns of regeneration of the NS, which is orchestrating the development of somatic and germline primordia.

#### 3.3.2. Coelom Wall and Muscular System 

The general character and the success of the regenerative processes in annelids largely depend on the structure of the mesodermal tissues of the body wall. A quick and efficient wound healing occurs in polychaetes and oligochaetes, with a well-developed metameric coelom. On the contrary, in *Arenicola* and *Terebella*, who have a common non-compartmentalized body cavity, injury leads to death because the coelomic fluid leaks out [128]. The body wall of leeches is enriched by loose connective tissue and an extracellular matrix, which is responsible for a particular pattern of reparative processes, in many ways reminiscent of scarring. In this case, various mesenchymal elements quickly form a wound-plug, but the restoration of the normal histological organization of the body wall, including the muscle layer, takes a very long time, over several months [129,130]. Thus, the loss of coelomic metamery, leading to a lesser segment autonomy, correlates with a weakening of the regenerative abilities in annelids [6].

In response to injury, the body wall performs both structural and regulatory functions. First of all, mesodermal tissues play a leading role in the formation of the regeneration blastema [10,21,131]. Distinct mesodermal cell types (i.e., muscular, myoepithelial, peritoneal, coelomocytes, parenchymal, and others) contribute differently to the blastema in different species, which reflects a significant diversity and an independent divergence of coelom tissues in the course of annelid evolution [132]. Secondly, the coelom functions as a cell source and a barrier to the migrating immunocompetent cells. It has been experimentally proven that the coelom wall and coelomocytes are essential elements of an early inflammatory response, including chemotaxis, migration, diapedesis, encapsulation, phagocytosis, and secretion of humoral mediators [129,133,134]. Thirdly, muscles and fibroblasts accumulate spare nutrients such as glycogen and fat, which are mobilized during the regenerative response [131,135]. Finally, transplantation experiments indicate that the body wall determines the polarity of the regenerating tissue [109] and that it also can induce parapodia morphogenesis at the border of positional discontinuity, i.e., in tissues with positional conflict [136]. These data suggest that the mesodermal derivatives in annelids may provide positional information, as they do in planarians and acoelomorphs [137]. The current experimental data indicate an overall importance of the coelom and muscles, but the hypothetical mechanisms of their involvement in regeneration should be detailed and verified with the use of new methods and new research objects.

**Figure 4:**
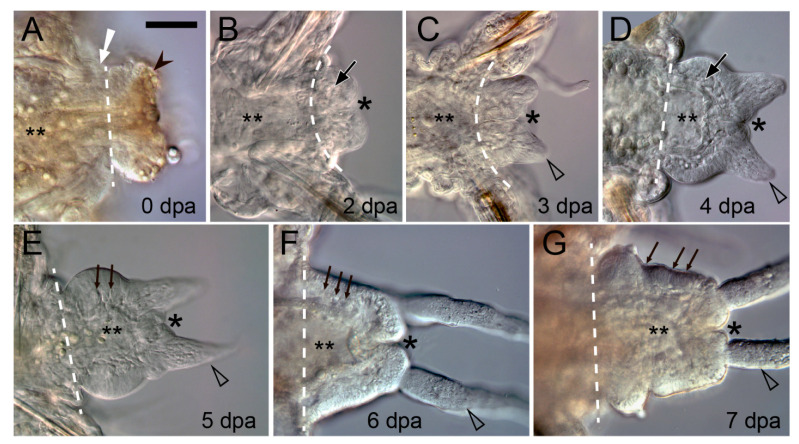
Posterior regeneration in a juvenile polychaete *Platynereis dumerilii* (Errantia, Nereididae). Optical frontal sections of live anesthetized (**A**,**F**,**G**) or fixed and glycerol-cleared (**B**–**E**) specimens 0 to 7 dpa; posterior end is to the right. Asterisk, anus; dotted line, plane of amputation; double asterisk, gut lumen; white arrow in (**A**), constriction of circular muscles; black arrowheads in (**A**), protruded and curled out gut; triangles in (**C**–**G**), forming pygidial cirrus; large arrows in (**B**,**D**), mesodermal cells of the regeneration blastema; small arrows, segmental boundaries in mesodermal (**E**,**F**) or ectodermal (**G**) tissues. Scale bar 50 μm.

The musculature has specific and very conservative functions in annelid regeneration. In response to an intersegmental transverse cut (amputation) the circular muscles in the vicinity of the wound contract immediately (Figure 2A and Figure 4A) [138,139,140]. This helps to reduce the wound surface, thus facilitating epithelialization. Interestingly, in injured leeches, contractile myofibroblast-like cells are involved both in constricting the wound edges and in retracting the pseudoblastema, “swallowing” the scar tissue deep into the parenchyma [32]. Another important aspect of the functioning of the circular muscles is the creation of a strictly defined autotomy plane. It is advantageous for reducing blood loss and eliciting a less acute immune response [141]. In oligochaetes that are capable of architomy, such as *Enchytraeus* and *Lumbriculus*, special muscles determine the plane of separation of body fragments [142,143], which is extremely important because it is essential for the correct polarization of the regenerate. Corrective autotomy occurs even in the case of artificial amputation; otherwise, e.g., if the musculature constriction is suppressed with anesthesia, a bipolar worm with two heads is formed [143]. During complete regeneration of segments, as in polychaetes and oligochaetes, the contracted muscles remain at the amputation level and do not shift away to the terminal end of the regenerative bud. The new-born muscle elements of the regenerate are certainly involved in the mechanical integration of the old and the new tissues.

The muscular system changes in the area of amputation very quickly. Damaged muscle fibers often degrade during wound healing [10,31,37,51,63,140]. Shedding of the contractile portion of myocytes has been described in detail in *Owenia* [37]. The resulting enucleated sarcolites degenerate in the coelom or end up phagocytized, which has also been documented for other species [10,31,51]. The degenerating muscles of an *Eisenia* amputee are dispersed within live muscle fibers [140]. Damaged and dying fibers of the ventral longitudinal muscles in *Nephtys* concentrate in the dorsomedial regions [63]. According to some authors [139,140,144], muscles predominantly die, although their dedifferentiation has been observed in *Owenia* and *Limnodrilus* [31,37]. An expression activation of multipotency markers *SOX2* and *H2B*, first in the circular and then in the longitudinal muscle layers in the wound zone of *Eisenia*, has been recently described [28]. The authors suggest this to be a sign that pluripotent stem cells appear in the muscle tissue. Nevertheless, this interpretation of the blastema precursors does not rule out the dedifferentiation mechanism [145]. Thus, out of all organ systems, it is the musculature of the terminal old segment that undergoes the most profound and diverse transformations after amputation.

The epimorphic processes of the muscle layer reconstruction have received much attention, in part due to the development of staining methods with labeled phalloidin and confocal microscopy [33,38,79,84,88,89,146,147]. Muscle precursors (mesodermal cells of the blastema) accumulate mainly in the ventral and lateral parts of the regenerative bud, between the intestine and the epidermis. A common rule of posterior regeneration is that muscles associated with pygidial structures are the first to differentiate [12]. For example, in *Alitta, Platynereis* and *Nephtys*, the anal sphincter is the first of the regenerated muscles differentiating in the pygidial coelomic wall (Figure 3A) [39,63,79]. At first (from 2 dpa in nereids), it consists of a few thin circular fibers at the posteriormost end of the regenerate. Later, the sphincter grows, connecting with longitudinal muscle fibers by 4–5 dpa [79]. In the absence of complex pygidial structures, as in oligochaetes, as well as during anterior regeneration, the longitudinal muscles differentiate faster than other fibers [12,56]. They are always continuously connected with the corresponding bundles and bands of the old segments, so that their development progresses in the proximodistal direction (Figure 3A). This characteristic pattern of ingrowth of longitudinal muscle fibers from the old segment is but a very rough reflection of the nature of morphological events, although some authors [12,38,56,88] consider it as an evidence in favor of the idea that longitudinal muscles have an independent source and mechanism of formation. An indirect confirmation of this possibility is the well-known plasticity of the differentiation and rearrangement of longitudinal muscles during stolonization and epitoky in polychaetes [148,149]. Nevertheless, several ultrastructural studies indicate that the muscles of the body wall regenerate from a common blastemal material [31,37,140]. The circular, transverse, and parapodial muscles develop later than the longitudinal ones; in posterior regeneration, an anterior-to-posterior gradient of their initiation and maturation is apparent [38,79,84,89,146]. Myofibers of myoepithelial cells of dissepiments, mesentery, and intestinal wall differentiate simultaneously with those of the somatic muscles. Thus, despite the significant progress in understanding the patterns of muscle differentiation, most issues concerning the origin, initiation, and histogenesis of contractile cells of the regenerate remain controversial.

So far, no targeted molecular genetic studies of muscle regeneration in annelids have been published. RNA-seq data on the earthworms *Eisenia* and *Eudrilus* indicate the presence of a large proportion of cells and genes associated with muscle differentiation in the regenerate [27,28]. Such marker genes with an enriched expression in the regenerative tissues are myosin heavy chains (paralogs of *MHC*, *Myosin-10*), tropomyosin (*TPM*), and obscurin (*UNC-89*). The expression of regulatory mesodermal markers during regeneration has been studied only in nereids (Figure 3C) [19,39,46,150,151]. The *twist* mRNA expression in *Platynereis* appears from 1 dpa, first at the site of the future anal sphincter (circular muscles of the pygidium), and a bit later in the segmental part of the regenerate. In *Alitta*, according to our preliminary data, *twist* is expressed in the wound from 8 hpa, and then in the blastemal cells, which give rise to the pygidial muscles, oblique muscles of the segments, and ventral medial longitudinal muscle. At the final stages of regeneration and during postlarval growth, the expression of *Avi-twist* was also detected in the mesodermal part of the growth zone. The expression of *prdm3/16* and FGF signaling components was revealed in the coelomic wall of the segments [39,46]. During regeneration, these markers are associated with the development of blood vessels and coelomic lining. Thus, the gene expression data indicate an early patterning of the mesodermal cells of the blastema and a broad scale of myogenesis.

#### 3.3.3. Digestive System 

The phenomenon of the gut de novo formation is still poorly characterized, especially on the molecular level. Both anterior and posterior regeneration are completed when the terminal regions of the digestive tract (the foregut and the hindgut, respectively) are restored, and the animals become capable of feeding and growing again. Different parts of the annelid gut have a different embryonic origin; the anterior gut and the hindgut derive from the ectoderm, while the midgut is an endodermal derivative. This complex origin of the digestive tract determines the level of gut plasticity during regeneration in annelids.

Following the constriction of circular body muscles at the initial stage of regeneration, the edges of gut epithelia severed upon transverse amputation typically heal by fusing to each other [4,6,10,21] (Figure 1). This produces a blind end of gut and a regeneration bud with no opening. In this case, the mouth or anus is formed de novo at the later stages. However, during posterior regeneration in sabellids and nereids, the severed edges of the epidermis fuse directly to the severed edges of the gut epithelium. In this case, the anus is restored directly by the wound healing process [34,79,131]. The same mechanism is used to close the wound at the posterior body end in the oligochaete *E. japonensis* [152]. A slight extrusion of the gut tissue with the following retraction and fusing of the severed edges has been described in *Dorvillea bermudensis* [43]. Thus, an injury of the posterior part of the intestine can be healed in two ways, suggesting that the mode of gut healing and anus regrowth can vary, even within a species [6]. However, in non-clitellate annelids, the endodermal gut is restored by growth of the surviving intestine, while the ectodermal pharynx is typically formed de novo by invagination of the outer epithelium into the blastema [62]. 

During posterior “open” regeneration in nereids, the gut healing and anus regrowth may involve morphallactic remodeling. In both *P. dumerilii* and *A. virens*, homologues of the *foxA* gene, a pioneer transcription factor specific for gut development, start to be expressed in cells close to the amputation site already at the stage of the wound closure. This early expression in preexisting tissue indicates that the terminal part of the gut undergoes the morphallactic remodeling as well as suggests its possible important role as a signaling center [34].

Posterior regrowth of the gut tissues also appears to be associated with the proliferation of endodermal epithelial cells. Experiments on *P. dumerilii* and *Syllis malaquini* with incubation with EdU prior to amputation support the idea that the intestinal epithelium contains stem cells that are normally required for physiological regeneration of the intestinal epithelium [39,45]. Active cell divisions of gut cells following amputation have been documented by autoradiography and histology for sabellid worms [131]. A cell-tracing technique has confirmed that old intestine tissue is indeed involved in new gut tissue formation in *L. variegatus* [153]. 

Endodermal gut tissue may be of critical importance for successful posterior regeneration. In *Autolitus pictus*, *Procerastea halleziana*, and *Syllis sp.*, regenerating fragments containing the head segments with only the ectodermal foregut (pharynx) cannot regenerate the lost posterior segments [62]. On the other hand, a regrowth of the intestine (endodermal origin) posteriorly from the foregut region (ectodermal origin) has been recently reported for syllid *S. malaquini* [45]. This result indicates that cell transdifferentiation might take place when the fragment contains no endodermal tissues from which the gut can be restored, as it was previously suggested [154,155]. 

During anterior regeneration, the wound healing process leads to the fusing of the severed intestine edges and the formation of the blind gut. Actively proliferating cells of the blastema fill the space between the blind gut end and the outer epithelium. The tip of the gut starts to grow ventrally and often extends until it contacts the VNC. The restored buccal cavity appears to originate from an invaginated outer ectoderm at the wound site. Thus, the oral opening is formed de novo. Eventually, the inwardly growing new foregut connects to the existing part of the intestine [20,38,62,131].

Formation of the pharynx from the invaginated outer ectoderm can occur within a specific range along the anteroposterior axis. For example, in *A. pictus*, the success of anterior regeneration correlates with the ability to restore the pharynx within only a few amputated anterior segments. Thus, de novo formation of the pharynx may play a key role in the anterior regeneration in annelids, determining the number of anterior regenerated segments [156].

In clittellates, the gut regrowth shows a more variable pattern during anterior regeneration than in non-clitellate annelids. In *E. foetida*, *T. rivulorum*, *L. variegatus*, and *E. japonensis*, the pharynx is derived from the old intestine of the endodermal origin. Nevertheless, even in this case, both the mouth and the buccal cavity originate from an invaginated outer ectoderm at the wound site [62]. 

In *L. variegatus*, cells of the existing gut tissue move within the epithelial layer and take part in the new pharynx development. These cells express the serine proteases that might provide local remodeling of the extracellular matrix, which ensures the migration of cells into the blastema, as well as the ingrowth of the nerve endings there. Inhibition of serine protease activity leads to the loss of regenerative ability [153]. Interestingly, the severing of the gut at the site of the wound is critically important for initiating blastemal outgrowth in *Tubifex* [10]. Moreover, endodermal cells were found in the inner blastema during the anterior regeneration of several annelid species [6,45].

De novo gut formation from two independent sources has been described for anterior regenerates of the clitellate *P. leidyi*. The inner blastema gives rise to the new pharynx, while the mouth originates from the invaginated surface ectoderm at the wound site [38]. During the stage of blastema growth, the deep cells start to express two *otx* homologues (*Pl-Otx1* and *Pl-Otx2*) during both anterior regeneration and asexual reproduction by paratomic fission. At later stages, these genes continue to be expressed in the pharynx of the new foregut [157].

Importantly, significant morphallactic remodeling of the gut can occur during the anterior and the posterior regeneration in annelids. In *E. japonensis*, patterns of *tuba*, *mino*, and *horu* expression suggest the restoration of the original proportions of the digestive tube by morphallaxis within the body [158]. At the same time, in *Pristina* spp., only a part of the intestine located in the two–three old segments adjacent to the wound loses the cilia and undergoes transformation into a new stomach-like structure during anterior regeneration [4,38]. 

Thus, gut regrowth occurs in a generally similar way during posterior regeneration, but the mode of gut healing and anus regrowth can vary, even within a species. In contrast, anterior regeneration in annelids is highly variable. De novo formation of anterior parts of the digestive tracts by the cells from different germ layers indicates fundamental differences in the mechanisms of the anterior and the posterior regeneration of annelids.

#### 3.3.4. Gonad and Germ Cell De Novo Formation during Regeneration and Asexual Reproduction in Annelids 

Annelids can regenerate gonads during asexual reproduction and morphallactic and epimorphic regeneration [48,159]. In non-clitellate annelids, gametogenesis usually occurs in a large number of segments. In this case, the germ cells are either located directly in the tissues of the body (often in association with the bases of the parapodia), or are separated from the surrounding tissues by a thin periotneum. The gonads of clitellate annelids are localized in several anterior segments [10,48].

During paratomy in clitellate *P. leidyi*, gonads are restored in daughter zoids. This type of regeneration is ensured by the migration of *piwi*-positive cells from the maternal gonads to the paratomy zone [48]. On the other hand, during anterior regeneration in *P. leidyi*, morphallactic restoration of the gonads occurs due to the rearrangement of the old segments into genital ones [159]. Surprisingly, no migrating *piwi*-positive cells were detected during this process, which indicates de novo formation of the germ cell lineage during morphallaxis [158]. Thus, even within the same species, the cellular sources of gonad restoration may be different during different variants of postembryonic morphogenesis.

In *E. japonensis*, gonad recovery was documented during both epimorphic regeneration and asexual reproduction [53]. Again, this process is due to migration of the germ cell precursors along the VNC. These cells are usually diffusely located along the VNC and represent a reserve population of germ cells [52]. To note, *piwi* homologue is a marker of these cells, which distinguishes them from neoblasts. It was found that *piwi*-positive cells migrate into the 7th and 8th segments restored during anterior regeneration, where they take part in the new gonad development. It is noteworthy that the gonad restoration in both *E. japonensis* and *P. leidyi* has been described only in the case of anterior regeneration.

In contrast, the restoration of the gonads during posterior regeneration has been shown for the polychaete *C. teleta*, which, unlike *E. japonensis* and *P. leidyi*, reproduces only sexually [59]. In *C. teleta*, the *piwi* transcripts appear de novo in blastema cells, which indicates the absence of cell migration with the same expression feature. The authors have assumed that the germ cells are restored by dedifferentiation in this species, without the involvement of reserve stem cells. Interestingly, later it was shown that the somatic cell lines may be restored by undifferentiated cells migrating from the MPC cluster into the blastema in this species [40].

## 4. Conclusions

Annelids demonstrate impressive regenerative abilities. At the same time, the mode of healing, the cellular sources of regeneration, and their induction can vary, even within a species. Despite a long history of research on annelid regeneration, many important questions regarding the mechanisms of annelid regeneration remain obscure. Because regenerating tissues may be formed from preexisting pluripotent stem cells, or by de-differentiation or trans-differentiation of differentiated cells, the most fundamental question concerns the (stem) cell niche and its changes during annelid regeneration. The growing knowledge of regeneration in other animals suggests an important role of apoptosis in the induction of cell proliferation and the emergence of signals within the body that can significantly alter the epigenetic landscape and ensure regenerative ability. Nevertheless, the subtle mechanisms of regeneration initiation in annelids are poorly understood and require further investigation. Many conclusions regarding the cellular aspects of annelid regeneration are mainly based on indirect evidence and need to be verified. New approaches, such as comparative transcriptomics, single-cell sequencing, in vivo imaging with labeling of cells, and functional analysis, as well as new techniques of morphological studies, may bring about major advances in our understanding of the cellular and molecular mechanisms of regeneration in annelids.

## Figures and Tables

**Table 1:**
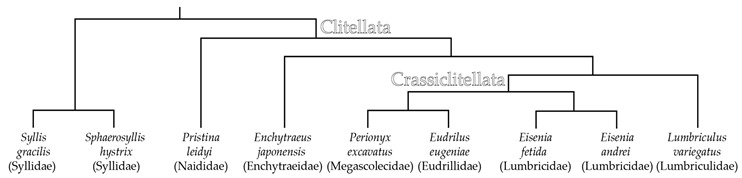
An overview of the quantitative results of transcriptomic data on the annelid regenerates. dpa, days after amputation; hpa, hours after amputation.

Sampling reference	[22]	[22]	[25]	[23]	[24]	[29]	[26]	[28]	[30]
anterior regeneration(AR) stages	1, 3, 6, 8 dpa	1, 5, 9, 12 dpa	mixed-stage regenerating and fissioning worms	mixed AR and PR samples (2, 3, 4, 11, 14, 16, 17, 18, 21, 22, 26, 27, 34, 38, 41, 47, 51, 53, 58 hpa)	mixed-stage (0.5, 1, 3, 6, 12, 18, and 24 hpa)	4, 6 dpa	not sampled	72 hpa (single-cell RNA-Seq)	24, 48, 72 hpa
posterior regeneration(PR) stages	1, 3, 6, 8 dpa	1, 5, 9, 12 dpa			not sampled	not sampled	15, 20, 30 dpa	0, 6, 12, 24, 48, 72 hpa (RNA-Seq)	not sampled
time of complete regeneration in the experiments, temperature	complete AR after 14 dpa, 14 °C	incomplete prostomium after 14 dpa, 14 °C	no data	4–5 dpa, 24 °C	no data, 22–24 °C	no data	no data, 22 °C	after 18 dpa, 25 °C	no data, 20 °C
technique	RNA-Seq	RNA-Seq	454 pyrosequencing	suppression subtractive hybridization	expressed sequence tags (ESTs)	RNA-Seq	RNA-Seq	single-cell RNA-Seq, RNA-Seq	RNA-Seq
number of identified/assembled sequences	526,860 contigs	315,224 contigs	111,201 unigenes		803 genes	105,193 contigs	125,896 contigs	up to 233,332 contigs	164,769 transcripts
number of specifically upregulated genes	1940 (AR) and 33 (PR) differentially expressed genes	4699 (AR) and 161 (PR) differentially expressed genes	no data	165	no data	3986	3589, 1887, 617 (15, 20, 30 dpa)	6048 differentially expressed genes	111
number of specifically downregulated genes			no data	no data	no data	6882	5124, 3612, 614 (15, 20, 30 dpa)		25

## Data Availability

Not applicable.
